# Sex differences in use of interventional cardiology persist after risk adjustment

**DOI:** 10.1136/jech.2008.077537

**Published:** 2008-12-03

**Authors:** N Nante, G Messina, M Cecchini, O Bertetto, F Moirano, M McKee

**Affiliations:** 1 Health Services Research Laboratory, University of Sienna, Sienna, Italy; 2 Regional Agency for Health Services of Piedmont, Piedmont Region, Turin, Italy; 3 Local Health Agency, Cuneo, Mondovì and Savigliano, Piedmont Region, Italy; 4 European Centre on Health of Societies in Transition, London School of Hygiene and Tropical Medicine, London, UK

## Abstract

**Background:**

Studies from several countries have documented gender disparities in the management of coronary artery disease. Whether such gender disparities are seen in Italy and, if so, whether they can be explained by factors such as age and severity of illness were investigated.

**Methods:**

77 974 Piedmontese patients, admitted between 1999 and 2002, with a primary diagnosis of myocardial infarction (ICD 410), angina (ICD 413), chronic ischaemia (ICD 414) and chest pain (ICD 786.5) were studied. The number of men and women undergoing surgical treatment was extracted and the male–female odds ratios calculated. Several risk factors and a risk adjustment technique (APR-DRG) were used to control for possible confounders. Backward stepwise multiple logistic regression was used to adjust for significant covariates.

**Results:**

Crude analysis demonstrated that gender is a discriminating factor in the probability of surgery (OR 2.11, 95% CI 2.04 to 2.19), with similar findings among those with each main diagnosis. The odds ratios decreased after adjustment for age, co-morbidity and disease severity but remained significant.

**Conclusions:**

Men and women admitted to hospitals in a region of northern Italy with a diagnosis of cardiovascular disease are treated differently and this cannot be explained by age or severity of disease.

Coronary artery disease is the single disorder most likely to kill women in developed countries.^1^ ^2^ There is now evidence from several studies, especially from the USA and the UK^3^^–^8 but also from Japan^9^ and several continental European countries^10^^–^13 that, compared with men with cardiovascular disease, women are less likely to receive interventional cardiac procedures. Yet this finding is not universal and some researchers have found no significant gender difference in utilisation of either medical therapies^14^^–^16 or surgical procedures.^8^ ^15^ ^17^ Where differences have been found, they have been documented at different stages in the process of accessing investigation and subsequent treatment.^18^

The contemporary relevance of these findings to Italy can, however, be questioned. First, given how the social meaning of gender is influenced by culture, it cannot be assumed that the phenomenon of gender disparity will be found everywhere. Second, some studies were undertaken when coronary revascularisation was still a relatively new procedure and when cardiovascular disease was still seen as essentially a male disease.^19^ Indeed, it has been argued that the modus operandi, both for investigation and for treatment of this disease, was developed primarily for men.^7^ ^20^ It is plausible that the subsequent expansion of provision could have redressed the observed gender imbalance, as has been seen to some extent in Finland.^13^

The aim of this study was to investigate whether, in Italy at the beginning of the twenty-first century, (1) there were gender disparities in the likelihood of being surgically treated for coronary artery disease and, if this is the case, (2) to ascertain the extent to which age, diagnosis, presence of other risk factors, severity of illness and risk of mortality may account for these differences.

## METHODS

Data on all discharges from every public and private hospital in the Italian region of Piedmont between 1 January 1999 and 31 December 2002 were obtained. Piedmont is one of the 20 administrative regions of Italy and, during those years, the population of 4 255 456 was 48% men. In the study period, the hospital network (public and private) comprised about 90 facilities.

Discharge data are collected routinely on all patients admitted to any Italian hospital, with details completed by trained staff (usually physicians or nurses). Each form includes (1) personal data: name, age, gender, nationality, place of residence; (2) diagnosis codes (up to six); (3) procedures undertaken (up to six); (4) length of stay; and (5) department of admission and discharge.

Data on diagnosis and procedures are coded using the International Classification of Disease, Ninth Revision, Clinical Modification (ICD-9-CM). Additionally, for each patient admitted in 2000 and in 2002 severity of illness and risk of mortality were calculated using the 3M APR-DRG classification system, V12. This proprietary software package assigns a value from 0 to 3 to each patient, corresponding to increasing risk. This score is generated using data on age and the presence of co-morbidities, so allowing a meaningful comparison of patients.

Among the 3 320 064 discharge forms collected between 1999 and 2002, we selected, for each year, all patients who were admitted with a primary diagnosis of myocardial infarction (ICD-9-CM code 410.XX), angina (code 413.X), chronic ischaemia (code 414.XX) and chest pain (code 786.5X).

We were interested in the probability of undergoing a first revascularisation; the factors influencing the decision to offer subsequent procedures may be different. Hence, we excluded all records subsequent to the one when patients underwent a revascularisation.

The choice of diagnoses was guided primarily by the desire to be consistent with previous papers.^21^ ^22^ However, we have also responded to criticism of some earlier papers by including chest pain (which, as with the other diagnoses, is analysed separately). This is because it has been argued that there is a systematic gender bias, in part explained by the tendency for presentation to be “atypical”, that leads to underdiagnosis of chest pain as being of cardiac origin in women.^8^ We do, however, recognise that this is a heterogeneous population, many of whom will not have cardiovascular disease.

Intervention was defined as the presence of codes for either percutaneous coronary intervention (ICD-9-CM code 36.0X) or coronary artery bypass (code 36.1X–36.2) in one of the six procedure fields of the discharge form.

An initial analysis tabulated the frequencies of each primary diagnosis, age group (⩽40, 41–50, 51–60, 61–70, 71–80 and ⩾81) and secondary diagnosis. As noted above, for 2000 and 2002, the 3M APR-DRG severity of illness (reflecting expected resource use) and risk of mortality were included. The next step was to calculate, for each variable, the percentage, for both men and women, who had been surgically treated.

When there was an association between gender and revascularisation, bivariate analysis was used to estimate the corresponding male–female odds ratios, with 95% confidence intervals, for being treated.

To reduce the influence of potential confounders adjustment was made for age group, secondary diagnosis of congestive heart failure (code 428.X) and diabetes mellitus (code 250.XX), which, from the literature, emerged as the quantifiable factors most likely to influence treatment decisions. For cases from 2000 and 2002, we were able to generate two models for each diagnosis, each containing a different variable derived from the APR-DRG system. These are severity of illness and risk of mortality. As they are reasonably closely correlated with each other, it was not thought appropriate to use them both in the same model.

Given the likely inter-relationships between potential explanatory variables, multiple logistic regression was used to determine the odds of undergoing revascularisation after adjustment for significant covariates, identifying the most parsimonious model. Thus, the regression used backward elimination. The initial model included all variables and those with the highest p values were eliminated at each step until only those that were significant remained.

The two datasets (with and without APR-DRG data) were independently analysed.

Data management was performed using Access 2003 whereas all the statistical procedures were carried out using EpiInfo V3.3.2 and STATA V8.0.

## RESULTS

A total of 77 974 cases fulfilled the inclusion criteria, equally distributed throughout the study period with a mean of 19 493 cases recorded each year (standard deviation (SD) 417), and women constituted around 32% each year. Over the whole study period 80.5% of patients were admitted only once, whereas readmissions accounted for 19.5% of cases.

The distribution of key variables within the cases meeting the inclusion criteria is shown in tables 1 and 2, with the numbers in parenthesis representing the percentages of patients undergoing surgical procedures. The length of stay variable, in the lowest rows, shows the mean of the hospitalisation days, for each diagnosis, and the corresponding SD.

**Table 1:** Number of hospitalised patients and percentage of operated

		1999					2000				2001				2002			
		Men			Women		Men		Women		Men		Women		Men		Women	
		Number		(%)	Number	(%)	Number	(%)	Number	(%)	Number	(%)	Number	(%)	Number	(%)	Number	(%)
Total		13202		(23.4)	6316	(12.4)	13266	(28.6)	6367	(15.7)	13402	(32.5)	6503	(19.3)	12722	(36.7)	6196	(20.7)
Diagnosis	Myocardial infarction	4624		(14.7)	2165	(7.2)	5170	(23.2)	2405	(11.4)	5220	(27.8)	2710	(17.3)	5121	(32.3)	2659	(17.1)
	Angina	2852		(20.8)	1197	(11.4)	2971	(33.4)	1329	(23.6)	2957	(37.1)	1253	(25.6)	2352	(39.9)	1027	(26.2)
	Chronic ischaemia	4317		(42.1)	2004	(24.5)	3668	(43.4)	1650	(24.7)	3884	(46.1)	1552	(30.0)	3968	(51.8)	1577	(35.3)
	Chest pain	1409		(0.1)	950	(0)	1457	(0.4)	983	(0.2)	1341	(0.5)	988	(0.1)	1281	(1.1)	933	(0.2)
Age group	⩽40	397		(16.6)	111	(9.9)	361	(19.4)	114	(12.3)	432	(20.8)	92	(12.0)	338	(23.4)	86	(10.5)
	41–50	1397		(27.4)	263	(14.1)	1326	(33.2)	253	(17.4)	1272	(36.7)	232	(20.7)	1214	(42.3)	240	(22.5)
	51–60	3069		(28.0)	727	(15.8)	3140	(33.9)	792	(19.0)	2901	(38.9)	800	(23.0)	2758	(40.9)	685	(24.7)
	61–70	4413		(26.1)	1651	(19.0)	4407	(31.4)	1625	(23.8)	4470	(36)	1666	(27.8)	4118	(40.7)	1564	(29.5)
	71–80	3025		(19.8)	2084	(13.4)	3198	(24.6)	2097	(17.7)	3441	(28.6)	2297	(21.5)	3342	(34.4)	2118	(23.1)
	>81	901		(4.1)	1480	(1.8)	834	(5.8)	1486	(2.1)	886	(7.8)	1416	(3.9)	952	(12.3)	1503	(6.5)
Second diagnosis	Congestive heart failure	Yes	671	(11.0)	470	(5.1)	688	(13.2)	554	(6.3)	701	(16.0)	520	(8.8)	673	(16.2)	600	(7.2)
		No	12531	(24.1)	5846	(13.0)	12578	(29.4)	5813	(16.6)	12701	(33.4)	5983	(20.2)	12049	(37.8)	5596	(22.1)
	Diabetes mellitus¶	Yes	1205	(22.6)	871	(13.6)	1498	(31.4)	994	(19.6)	1614	(31.5)	1100	(20.4)	1394	(32.8)	1021	(20.5)
		No	11997	(23.5)	5445	(12.2)	11768	(28.2)	5373	(14.9)	11788	(32.6)	5403	(19.1)	11328	(37.1)	5175	(20.7)
APR-DRG Severity of illness	1	–		–	–	–	8909	(30.3)	3794	(18.6)	–		–	–	8441	(40.4)	3646	(25.4)
	2	–		–	–	–	3348	(24.4)	1852	(11.1)	–		–	–	3265	(28.2)	1846	(13.2)
	3	–		–	–	–	802	(28.9)	574	(12.0)	–		–	–	798	(35.5)	539	(15.2)
	4	–		–	–	–	206	(20.4)	147	(10.2)	–		–	–	218	(23.4)	165	(17.0)
APR-DRG Risk of mortality	1	–		–	–	–	10657	(31.2)	4568	(18.3)	–		–	–	10032	(40.5)	4356	(24.7)
	2	–		–	–	–	1749	(17.9)	1164	(9.8)	–		–	–	1814	(23.1)	1175	(11.7)
	3	–		–	–	–	359	(18.4)	245	(8.2)	–		–	–	408	(19.1)	333	(9.3)
	4	–		–	–	–	500	(16.2)	390	(7.4)	–		–	–	468	(22.7)	332	(11.4)

Myocardial infarction, 410.XX ICD9-CM; chest pain, 786.5X ICD9-CM; angioplasty, 36.0X ICD9-CM; angina, 413.XX ICD9-CM; congestive heart failure, 428.X ICD9-CM; coronary artery bypass, 36.1X-36.2X ICD9-CM; chronic ischaemia, 414.XX ICD9-CM; diabetes mellitus, 250.XX ICD9-CM.

**Table 2:** Length of stay (with SD) by diagnosis

		Mean		SD	Mean	SD	Mean	SD	Mean	SD	Mean	SD	Mean	SD	Mean	SD	Mean	SD
Length of stay	Myocardial infarction	10.1		7.1	11.2	9.6	9.4	6.8	11.3	10.0	8.5	7.2	10.1	9.4	8.1	6.6	9.6	9.9
	Angina	7.0		7.5	7.6	7.0	6.6	7.2	7.3	7.9	6.0	6.6	6.7	8.0	5.6	6.7	6.4	7.3
	Chronic ischaemia	9.9		24.1	12.7	21.1	8.9	17.8	13.8	18.8	8.1	15.6	12.7	18.1	8.5	14.1	12.1	17.3
	Chest pain	4.1		6.0	4.5	5.8	4.1	6.0	5.0	11.4	3.9	5.5	4.5	7.4	3.4	3.4	3.8	3.3

Female patients were, on average, older than male patients, respectively 71.4 (SD 12.5) and 64.2 (SD 12.0). Among those undergoing surgery, men had a mean age of 62.6 (SD 10.2) and women of 67.7 (SD 9.41) (a difference of 5.1 years; p<0.001), whereas among all those hospitalised who did not have revascularisation the figures were respectively 64.9 (SD 12.7) and 72.2 (SD 12.9) years old (a difference of 7.3 years; p<0.001).

There was an upward trend over time in the percentage of patients undergoing interventions, especially among those with diagnoses of angina or myocardial infarction, in which operation rates more than doubled, increasing respectively from 14.7% in 1999 to 32.3% in 2002 for men and from 7.2% to 17.1% for women in the same period.

Table 3 shows the crude and adjusted odds ratios (ORs) for revascularisation procedures among men compared with women, according to potential explanatory variables. Crude analysis demonstrated that, for all diagnoses combined, being male is an important discriminating factor in whether or not one undergoes revascularisation (OR 2.11, 95% CI 2.04 to 2.19, p<0.001) (table 4). Broadly comparable findings were obtained within each diagnostic category: myocardial infarction, OR 2.09 (95% CI 1.96 to 2.23); angina, OR 1.75 (95% CI 1.61 to 1.90); chronic ischaemia, OR 2.14 (95% 2.01 to 2.28); and chest pain, OR 3.95 (CI 1.52 to 10.24) (table 3).

**Table 3:** Crude and adjusted ORs for revascularisation in men compared with women

		Myocardial infarction (ICD-9: 410.XX)		Angina (ICD-9: 413.XX)		Chronic ischaemia (ICD-9: 414.XX)		Chest pain (ICD-9: 786.5X)	
		OR (95% CI)	p Value	OR (95% CI)	p Value	OR (95% CI)	p Value	OR (95% CI)	p Value
Crude		2.09 (1.96 to 2.23)	<0.001	1.75 (1.61 to 1.90)	<0.001	2.14 (2.01 to 2.28)	<0.001	3.95 (1.52 to 10.24)	0.0023
All years adjusted for	Age band	1.35 (1.26 to 1.45)	<0.001	1.59 (1.46 to 1.72)	<0.001	1.49 (1.39 to 1.59)	<0.001	4.07 (1.60 to 10.35)	0.0014
	Secondary diagnosis of diabetes	2.07 (1.94 to 2.21)	<0.001	1.77 (1.64 to 1.92)	<0.001	2.13 (2.0 to 2.26)	<0.001	3.96 (1.54 to 10.16)	0.002
	Secondary diagnosis of heart failure	1.99 (1.86 to 2.13)	<0.001	1.74 (1.61 to 1.89)	<0.001	2.13 (2.0 to 2.27)	<0.001	3.95 (1.52 to 10.25)	0.0023
Crude		2.28 (2.08 to 2.50)	<0.001	1.73 (1.55 to 1.94)	<0.001	2.15 (1.97 to 2.35)	<0.001	3.52 (1.20 to 10.30)	0.0014
2000 and 2002 adjusted for	Age bands	1.47 (1.34 to 1.62)	<0.001	1.58 (1.41 to 1.76)	<0.001	1.52 (1.38 to 1.67)	<0.001	3.92	0.0076
	Secondary diagnosis of diabetes	2.26 (2.06 to 2.48)	<0.001	1.77 (1.58 to 1.97)	<0.001	2.14 (1.95 to 2.34)	<0.001	3.53 (1.22 to 10.24)	0.0132
	Secondary diagnosis of heart failure	2.15 (1.96 to 2.36)	<0.001	1.73 (1.55 to 1.93)	<0.001	2.14 (1.95 to 2.33)	<0.001	3.51 (1.20 to 10.31)	0.0146
	Severity of illness	1.99 (1.81 to 2.18)	<0.001	1.76 (1.57 to 1.97)	<0.001	2.12 (1.94 to 2.32)	<0.001	3.32 (1.12 to 9.76)	0.0211
	Risk of death	2.01 (1.83 to 2.20)	<0.001	1.73 (1.55 to 1.93)	<0.001	2.08 (1.90 to 2.27)	<0.001	3.49 (1.19 to 10.24)	0.0152

**Table 4:** Models of the relationship between probability of intervention and gender adjusted for possible confounders

Year	Pathology	Starting model	OR (95% CI)	p Value	Final model	OR (95% CI)	p Value
1999–2002	All diagnoses		2.11 (2.04 to 2.19)	<0.001			
	Myocardial infarction	hrtfail+diab+clsage	1.33 (1.24 to 1.43)	<0.001	hrtfail+diab+clsage	1.33 (1.24 to 1.43)	<0.001
	Angina		1.61 (1.48 to 1.75)	<0.001		1.61 (1.48 to 1.75)	<0.001
	Chronic ischaemia		1.48 (1.39 to 1.58)	<0.001		1.48 (1.39 to 1.58)	<0.001
	Chest pain		4.30 (1.65 to 11.23)	0.003	–	3.95 (1.52 to 10.24)	0.002
2000 and 2002	All diagnoses		2.18 (2.06 to 2.29)	<0.001			
	Myocardial infarction	hrtfail+diab+clsage+sevill	1.43 (1.30 to 1.58)	<0.001	clsage+sevill	1.43 (1.30 to 1.58)	<0.001
		hrtfail+diab+clsage+riskmort	1.44 (1.31 to 1.59)	<0.001	clsage+riskmort	1.45 (1.31 to 1.59)	<0.001
	Angina	hrtfail+diab+clsage+sevill	1.55 (1.38 to 1.12)	<0.001	clsage+sevill+hrtfail	1.56 (1.39 to 1.75)	<0.001
		hrtfail+diab+clsage+riskmort	1.61 (1.44 to 1.80)	<0.001	hrtfail+diab+clsage+riskmort	1.61 (1.44 to 1.80)	<0.001
	Chronic ischaemia	hrtfail+diab+clsage+sevill	1.53 (1.39 to 1.69)	<0.001	clsage+hrtfail+sevill	1.54 (1.40 to 1.69)	<0.001
		hrtfail+diab+clsage+riskmort	1.52 (1.38 to 1.67)	<0.001	clsage+hrtfail+riskmort	1.53 (1.39 to 1.68)	<0.001
	Chest pain	hrtfail+diab+clsage+sevill	3.69 (1.24 to 10.69)	0.019	–	3.52 (1.20 to 10.30)	0.022
		hrtfail+diab+clsage+riskmort	3.88 (1.31 to 14.47)	0.014	–	3.52 (1.20 to 10.30)	0.022

hrtfail, congestive heart failure; diab, diabetes mellitus; clsage: age group; sevill, severity of illness; riskmort, risk of mortality.

In all cases, the gap narrows when adjusted for age, but remains highly significant for all diagnostic categories. Adjustment for the presence of heart failure or diabetes makes a relatively small difference. In the subset of data with additional information on severity, adjustment again makes relatively little difference.

Table 4 shows the fully adjusted model, before and after backward elimination of variables starting with age group and presence or absence of diabetes or heart failure, plus, for 2000 and 2002, the two severity scores. In the initial analyses, using all 4 years of data, all three variables were retained, except for chest pain (which had the fewest numbers and the least likelihood of co-morbidity), in which they were all eliminated. In the restricted dataset from 2000 and 2002, among those with myocardial infarctions the two final models retained only age and severity or age and risk of mortality, suggesting that the presence of heart failure or diabetes as a factor that would reduce the probability of intervention is viewed similarly in men and women. In contrast, heart failure (and diabetes for those with angina after adjustment for mortality risk) do seem to have a different meaning in men and women with angina or chronic ischaemia. Chest pain does not seem to be influenced by any of the studied variables, although this is likely to reflect both the low numbers of cases, with some age groups containing no patients, and the low rate of co-morbidity.

Comparing the crude male–female ORs (table 3) with those in the fully adjusted models (table 4), it can be seen that, with the exception of the small number of patients with a diagnosis of chest pain, inclusion of all significant correlates reduces the gender gap (myocardial infarction 2.09 to 1.33; angina 1.75 to 1.61; and chronic ischaemia 2.14 to 1.48), but in all cases the greater probability of intervention in men remains highly statistically significant. Similar results were obtained using the subset of 2000 and 2002 data, when additional severity measures were included.

## DISCUSSION

In the early twenty-first century, Italian women remain less likely than men to undergo surgical procedures for coronary artery disease. This inequity is consistent with previous research undertaken in the USA, the UK and elsewhere. However, this study goes beyond many of the earlier studies by showing that the disparity persists after taking account of differences in severity of illness. We believe that this is particularly relevant as earlier research has given rise to the term “Yentl syndrome”,^23^ named after the film in which a woman disguises herself as a man to become a rabbi. It is suggested that “Once a woman showed that she was just like a man, by having severe coronary artery disease …, then she was treated as a man would be”. Thus, we would expect that the ORs would narrow markedly after adjustment for severity. There was some narrowing but, even after the inclusion of all significant variables, including sophisticated measures of disease severity, women were still less likely to undergo revascularisation.

It is beyond the scope of this study, which is based on administrative data, to explain why these inequities exist. Authors of previous studies proposed two sets of hypotheses to explain these gender differences. The first focuses on patient preferences. This is based on the premise that some women do not see themselves as candidates for revascularisation because they do not consider themselves to be at risk of coronary heart diseases. A study addressing this issue reported that all the women involved expressed surprise when informed of their diagnosis. The most obvious consequence of this finding is that, if a woman does not see herself as being at risk of cardiovascular disease, she will tend to interpret symptoms as being due to other reasons.^24^ It has also been reported that women are more likely than men to decline a major procedure.^20^ ^25^

Another factor is that women may present with symptoms that are “atypical”, which has been defined largely on the basis of studies among men. It has been found that women experiencing an acute myocardial infarction are more likely than men simultaneously to experience gastrointestinal symptoms such as indigestion,^26^ while it has also been suggested that there may be difficulty distinguishing some forms of angina from breast tenderness secondary to cyclical hormonal changes in premenopausal women or to hormone replacement therapy in postmenopausal women.^27^ Another study concluded that, while there were no major differences in the language used by women and men to report pain, women may localise it differently and may be more likely to report supplementary symptoms.^8^ We tried to consider this issue, at least partially, by including patients initially diagnosed as having chest pain rather than a specific cardiac cause. Even in this much broader group of patients, women continue to be less likely to be operated on.

The second group of hypotheses focuses on decisions made by physicians. There is now clear evidence that the gender of a patient may influence the response of physicians.^10^ Writers from a feminist perspective have argued that this reflects perceptions of the value that each gender contributes to society, with some male physicians believing that men contribute more than women.^28^ It has also been suggested, as noted above, that bias may arise from the use of diagnostic criteria developed and validated on men, which may thus be less sensitive and specific in women,^19^ so potentially misleading the diagnostician. For instance, women presenting with chest pain are less likely to have a positive exercise test and, among those who do have a positive exercise test, women are still less likely to have coronary artery disease.^29^ In addition, some studies suggest that women have more complications and benefit less following surgery.^20^

Unfortunately, it is not possible to test these hypotheses using these data. This would require other techniques such as ethnography or decision–analysis methods that could explore the decision-making processes adopted by patients and physicians. Nor is it possible to eliminate entirely some forms of artefact. There may also be valid clinical considerations that were undocumented in the clinical records, but these would require a prospective study which would be fraught with methodological and ethical problems. Finally, we cannot exclude the possibility that relevant co-morbidities and contraindications were inadequately documented by medical staff. It is, however, unlikely that there would be selective differences in recording by gender.

Our study exhibits both strengths and weaknesses. A major strength is that retrospective analysis of patients’ records ensured that clinical practice was not influenced by the research process, thereby biasing findings, but, on the other hand, our findings might be subject to the problems known to affect any study using routine information, such as incompleteness of clinical coding, data inaccuracies and failure to link episodes to individuals. Nevertheless, we believe that these risks are minimised by the presence in each facility of strict quality control systems monitoring the quality of data collection, albeit that the diligence with which these processes are undertaken reflects the need to monitor financial rather than clinical performance. Although we were able to remove all details of people who had records of previous revascularisation within the dataset, we cannot exclude the possibility that a small number may have undergone treatment either elsewhere or before the study years. It is, however, difficult to see, given the small numbers likely to be involved, how this could materially affect the results.

A second advantage is the inclusion of procedures in both public and private hospitals, especially in light of a recent study from the UK showing how private sector provision exacerbates social inequalities in access to care.^30^

A third advantage of our study is the inclusion of two measures of risk adjustment. In our study, men and women differ significantly in age. This is to be expected, given that coronary artery disease presents earlier in men,^31^ but could introduce a bias into the results. In calculating the severity of illness rank, the APR-DRG software takes into account not only relevant co-morbidities but also the interaction between these co-morbidities and age or the principal diagnosis.^32^ This is particularly useful as this procedure allows a meaningful and fairer comparison of the two groups.

A disadvantage is that we are able to look only at patients once they have reached hospital. We cannot exclude the possibility that women might be disadvantaged further by failure to refer them for investigation. This is less likely in cases of myocardial infarction but is certainly possible for angina and chronic ischaemia.

This study has found a substantial gender disparity in the probability of undergoing revascularisation among patients with known cardiovascular disease in a large Italian region. This cannot be accounted for by documented differences in age or severity of illness. This is, to our knowledge, the first time that this phenomenon has been documented in Italy. Furthermore, despite evidence of disparities from elsewhere, we are unaware of any significant discussion about this issue in Italy, either among patient or professional groups. While more research is clearly needed to understand these observations better, we hope that this study will at least enable the discussion to begin.

**What is already known on this subject**

Previous studies documented gender differences in the likelihood of being treated surgically for ischaemic heart disease. Many researchers suggested that women are less likely to be operated because they usually show a less severe picture. This phenomenon, known as the Yentl syndrome, implies that, once a woman assumes certain characteristics associated with men (such as classical clinical presentation), she will receive the same treatment.

**What this study adds**

Our results, based on a very large dataset, confirm gender differences for a number of cardiac-related diagnoses. Furthermore, we find that inequalities persist after adjustment for severity of illness and risk of mortality.
